# Genetic diversity of *Bemisia tabaci* species colonizing cassava in Central African Republic characterized by analysis of cytochrome c oxidase subunit I

**DOI:** 10.1371/journal.pone.0182749

**Published:** 2017-08-15

**Authors:** Brice Kette Tocko-Marabena, Semballa Silla, Christophe Simiand, Innocent Zinga, James Legg, Bernard Reynaud, Helene Delatte

**Affiliations:** 1 Laboratoire des Sciences Biologiques et Agronomique pour le Développement (LBSAD), Université de Bangui, Bangui, Centrafrique; 2 CIRAD, UMR PVBMT, Pôle de Protection des Plantes, Saint-Pierre, Île de la Réunion, France; 3 Université de la Réunion, UMR PVBMT, Pôle de Protection des Plantes, Saint-Pierre, Île de la Réunion, France; 4 IITA-Tanzania, Dar es Salaam, Tanzania; Zhejiang University, CHINA

## Abstract

After 2007, upsurges of whiteflies on cassava plants and high incidences of cassava diseases were observed in Central African Republic. This recent upsurge in the abundance of *Bemisia tabaci* (Gennadius) (Hemiptera: Aleyrodidae) was directly linked to serious damage to cassava crops resulting from spread of whitefly-borne cassava mosaic geminiviruses (CMGs). There is currently very little information describing whitefly populations on cassava and associated crops in Central African Republic. The current study aimed to address this gap, and to determine whether the increasing damage associated with *B*. *tabaci* whiteflies was the consequence of a new invasion, or an upsurge of a local population. The molecular genetic identification and phylogenetic relationships of 898 *B*. *tabaci* adult individuals collected from representative locations (54) throughout CAR were determined based on their mitochondrial cytochrome oxidase I sequences (mtCOI). Field and ecological data were also collected from each site, including whitefly abundance, CMD incidence, host plants colonized by *B*. *tabaci* and agro-ecological zone. Phylogenetic analysis of the whitefly mtCOI sequences indicated that SSA1 (-SG1, -SG2), SSA3, MED, MEAM1 and Indian Ocean (IO) putative species occur in CAR. One specific haplotype of SSA1-SG1 (SSA1-SG1-P18F5) predominated on most cassava plants and at the majority of sites. This haplotype was identical to the SSA1-SG1 Mukono8-4 (KM377961) haplotype that was recorded from Uganda but that also occurs widely in CMD pandemic-affected areas of East Africa. These results suggest that the SSA1-SG1-P18F5 haplotype occurring in CAR represents a recent invasive population, and that it is the likely cause of the increased spread and severity of CMD in CAR. Furthermore, the high mtDNA sequence diversity observed for SSA1 and its broad presence on all sites and host plants sampled suggest that this genetic group was the dominant resident species even before the arrival of this new invasive haplotype.

## Introduction

Cassava (*Manihot esculenta* Crantz, Euphorbiaceae) is a major staple crop in sub-Saharan Africa due to its high calorie content, low production cost and ability to adapt to different soil types and climatic conditions, most particularly in central Africa [1]. For example, in Central African Republic (CAR), annual production of cassava is approximately 2.4 million tons of fresh tuberous roots, equivalent to 600,000 t of chips, ten times higher than maize and groundnuts [2]. Production of cassava in Africa is currently constrained by pests and diseases, the most significant of which are cassava mosaic disease (CMD) and cassava brown streak disease (CBSD) [3, 4] and annual losses to CMD alone have been estimated at more than US$ 1 billion [5]. CMD occurs wherever cassava is grown in Africa, and is caused by nine cassava mosaic geminiviruses (CMGs) (family Geminiviridae, genus *Begomovirus*) [6] occurring singly or in mixed infections. The disease is known to be more severe where *African cassava mosaic* virus (ACMV) occurs in mixed infection with one of the several *East African cassava mosaic* virus (EACMV) -like viruses [7, 8]. Yield losses attributed to CMD were minor to moderate for many years, following the first outbreaks occurring during the 1920s-30s. Nevertheless it became much more damaging following the development of an unusually severe form of the disease in Uganda that spread to neighboring parts of East and Central Africa and became a regional pandemic [9]. CBSD is known to be caused by two plant RNA viruses, *Cassava brown streak virus* (CBSV) and *Ugandan cassava brown streak virus* (UCBSV) (family Potyviridae; genus *Ipomovirus*), occurring alone or in co-infection. This disease has recently affected much of the Great Lakes Region of East and Central Africa [10–12].

In CAR, previous disease surveys conducted in seven areas during 2005 and 2006 revealed CMD incidences (associated with ACMV and EACMV-UG) ranging from 67 to 97%, with tuberous root yield losses estimated at 49% [13, 14], but no CBSD was recorded. The common key factor driving both diseases spread is the occurrence of super-abundant populations of the whitefly, *Bemisia tabaci* (Gennadius) (Hemiptera: Aleyrodidae), causing not only physical damage to cassava but transmitting CMGs [15, 16]. Both biological and genetic evidence suggest that these populations of insects have changed in a way that has enhanced their adaptation to cassava [17–19]. The whitefly *B*. *tabaci* is a complex of genetically distinct cryptic species, with 35 putative cryptic species that have so far been identified worldwide [20–22]. Although major studies on *B*. *tabaci* have been performed in East and West Africa, little is known about the distribution and frequency of the various species in the countries of Central Africa. So far, the sub-Saharan African clade has been reported in this region [19] (which includes the sub-Saharan species together with their genetic groups called: SSA1-SG1, SSA1-SG2, SSA1-SG3, SSA1-SG4, SSA2 and SSA3). However, for the Central African Republic, the frequency of the species on cassava or other associated crops, and their genetic sub-divisions remain largely unknown.

The main objective of this study was to provide the first detailed description of the diversity of *B*. *tabaci* species in CAR on several host plant species. This will provide a good basis for answering questions about species dynamics in relation to super-abundance, virus diseases, as well as providing valuable information for developing management programs.

## Materials and methods

### Study area

This study was conducted in CAR during the months of February 2008 and 2013. CAR is located in the central part of the African continent, and bordered by 5 countries (Cameroon, Chad, Sudan, Republic of Congo and Democratic Republic of Congo). The climate is tropical with two seasons: the dry season from November to April and the rainy season from May to October.

### Whitefly sampling

Adult *B*. *tabaci* individuals were collected from the shoot tips of 3–5 month old cassava plants and weeds during surveys of 14 district areas (54 localities) distributed in three agro-climatic zones where cassava is cultivated: Guinean Forest (GF), Sudano-Ubanguian (SU) and Sudano-Guinean (SG) zones (S1 Table). Host-plants were selected along two diagonal transects across each cassava field, and whitefly abundance was recording according to the method described in [23]: whitefly abundance was determined as the total number of adults from the topmost five leaves of each of the plants of the diagonal surveyed. Altitude, longitude, latitude, abundance of whiteflies, and CMD incidence were recorded in each field, as well as geo-coordinates using a global positioning system (GPS) handset. Immediately following collection, the adults were preserved in 90% alcohol, and stored in a freezer at -20°C following survey completion. The number and distribution of sampling sites differed from year to year and between areas. In all cases, *B*. *tabaci* adults were collected from several cassava plants per sampled field and a single sample tube of at least 32 insects was collected from each sampled field, hereafter referred to as a site. A total of over 5000 whiteflies were collected during the dry seasons of 2007–2008 and 2013. From this total, 898 individuals were further processed in this study, of which 98 from 9 sites from 2007–2008 (mostly in the Bangui area) and 800 from all the other sites sampled in 2013. Samples were collected from all major cassava-growing areas of CAR. Adults were morphologically sexed under a binocular (x40) stereomicroscope [24] before DNA extraction. Host plants of the sampling period of 2013 included one weed species (*Sida acuta*) and six cultivated plants (groundnut: *Arachis hypogaea*, tomato: *Solanum lycopersicum*, eggplant: *Solanum melongena*, cassava: *Manihot esculenta*, sweet potato: *Ipomoea batatas*, and cotton: *Gossypium* sp.*)*, whereas only cassava were sampled in 2007–2008 (S1 Table).

### Field data analysis

Data analysis were performed with the software R (version 3.2.2 Development Core Team, 2015). The effect of agro-climatic zones was tested on whitefly abundance (assuming a Poisson distribution), using a Generalized Linear Model with a likelihood ratio test (Chi-square test or Fisher test in case of over-dispersion), and Tukey’s pairwise mean comparison test, as described in detail previously [25]. The distribution of whitefly on each host plant such as cassava cultivars and sites was also tested.

### Ethics statement

No specific permits were required for this study, as it did not involve human participants and/or tissues, vertebrate animals, or embryos. Whiteflies are not considered as an endangered or protected species according to the IUCN criteria. The sampling was carried out on private lands (i.e. fields), and for each location the owner of the land gave permission to conduct the whitefly sampling on the site.

### DNA extraction

Only females were used in this study. Each field-captured whitefly was incubated in 25 μL extraction buffer [50 mmol/KCl, 10 mmol/L Tris-base (pH 8), 0.45% IGEPALCA-630, 0.45% Tween 20 and 500 mg/mL proteinase K (Sigma)] at 37°C for 15h, then a last step of incubation was performed at 90°C for 10 min. Extracts were briefly centrifuged between each step. A volume of 35 μL of pure HPLC water (CHROMASOLV®, Sigma-Aldrich) was added, and extracts were finally stored at -20°C until use.

### Mitochondrial DNA amplification and analysis

At least fifty individuals were chosen from each sample. Mitochondrial COI (mtCOI) sequences were obtained by PCR amplification with the general primer set C1-J-2195 (5′-TTGATTTTTTGGTCATCCAGAAGT-3′) and L2-N-3014 (5′-TCCAATGCACTAATCTGCCATATTA) [26]. The PCR was conducted in a final volume of 20μL, 2x Type-it master mix (QIAGEN), 20μmol/L of each primer, and 10 ng of insect DNA extract. A first step of denaturation at 94°C for 5 min was followed by 40 cycles at 94°C for 20sec, 52°C for 30sec, with a final elongation step for 10min at 72°C. Each PCR product was sequenced (Macrogen Inc., Sequencing Service, Korea). DNA sequences produced in this study were identified using the BLASTn algorithm of GenBank (http://www.ncbi.nlm.nih.gov) and submitted to GenBank. Eighteen other mtCOI sequences were obtained from public sequence databases using the Tax browser (http://www.ncbi.nlm.nih.gov/Taxonomy/taxonomyhome.html). Multiple sequence alignments were constructed using the CLUSTALW-based alignment tool available in MEGA version 6 [27] and by manual editing.

### Genetic diversity and network analysis

The full dataset comprised 898 sequences, of which 587 were trimmed to a length of 504 nucleotides (nt) and 298 were trimmed to 450 nt. This second group of shorter sequences was only used for putative species diagnostic purposes (used for the overall distribution in CAR and host plant association) and not included in the sequences analysis.

A minimum-spanning tree was built using the software Phyloviz V. 1.0 (http://www.phyloviz.net) [28] in order to display the relationships between haplotypes and to assess mutational steps between them. A new data set was created on Phyloviz using only the 74 unique sequences retrieved among the 587 trimmed sequences.

### Tests for neutrality

Four different tests of selective neutrality and population stability were performed with DnaSP software [29]: Tajima’s D test, Fu and Li’s D* and F* tests, and the MacDonald-Kreitman test [30]. To infer the long-term demographic history of the populations, the R2 statistic was calculated. That measure is based on the difference between the number of singleton mutations and the average number of nucleotide differences among sequences [31]. The significance of R2 was evaluated by comparing the observed value to the distribution of R2s simulated under the standard coalescent, using the empirical population sample size and the observed number of segregating sites implemented in the software. Those neutrality tests were performed on three different population groups, comprising three different haplotypes: Med, SSA1-SG1 and SSA1-SG2.

### Phylogenetic analysis

Sequences were loaded into CLUSTALW with other close reference sequences from different species of *B*. *tabaci* from the GenBank database for sequence alignments (Table 1). To infer relationships between the different populations sampled, the 74 unique sequences of a standard 504 bp mtCOI fragment were used to generate the phylogenetic tree. An initial approximate maximum likelihood phylogeny for large alignments of the selected sequences was built, under the General Time Reversible model of nucleotide substitutions and varying substitution rates across sites (GTR+nt), with the software FastTree v2.1.7 [32]. Branch support was calculated by Shimodaira-Hasegawa-like local branch support (SH-like test), as implemented in FastTree. Trees were visualized with FigTree (V1.4.2) [33]. The 74 unique haplotype sequences were submitted to the EMBL database.

**Table 1:** *Bemisia tabaci* mtCOI sequences used to construct the phylogeny and haplotype network retrieved from Genbank.

Genbank references	Host plants	Authors	Sequence name	Species or putative species name
HQ908639	Marrow	[34]	B2-6	MED (ASL)
HQ908651	Cassava	[34]	To2-1	SSA3
KM407142	Cassava	[35]	UG_mukono1-7	SSA2
KM377899	Cassava	[35]	5–2.1_SSA1-SG2	SSA1-SG2
KM377961	Cassava	[35]	UG_Mukuno8	SSA1-SG1
KM377922	Cassava	[35]	NG_Oyo_state_3	SSA1-SG5
EU760759	Poinsettia	[36]	Reunion4	Indian Ocean
KU242421	Tomato	Direct submission	TO26	MEAM1
KF734668	Cassava	[37]	*Bemisa afer*	*Bemisa afer*
KM377969	Cassava	[35]	*UG_Ssanji*	SSA1-SG3

## Results

### Whitefly abundance

**Fig 1:**
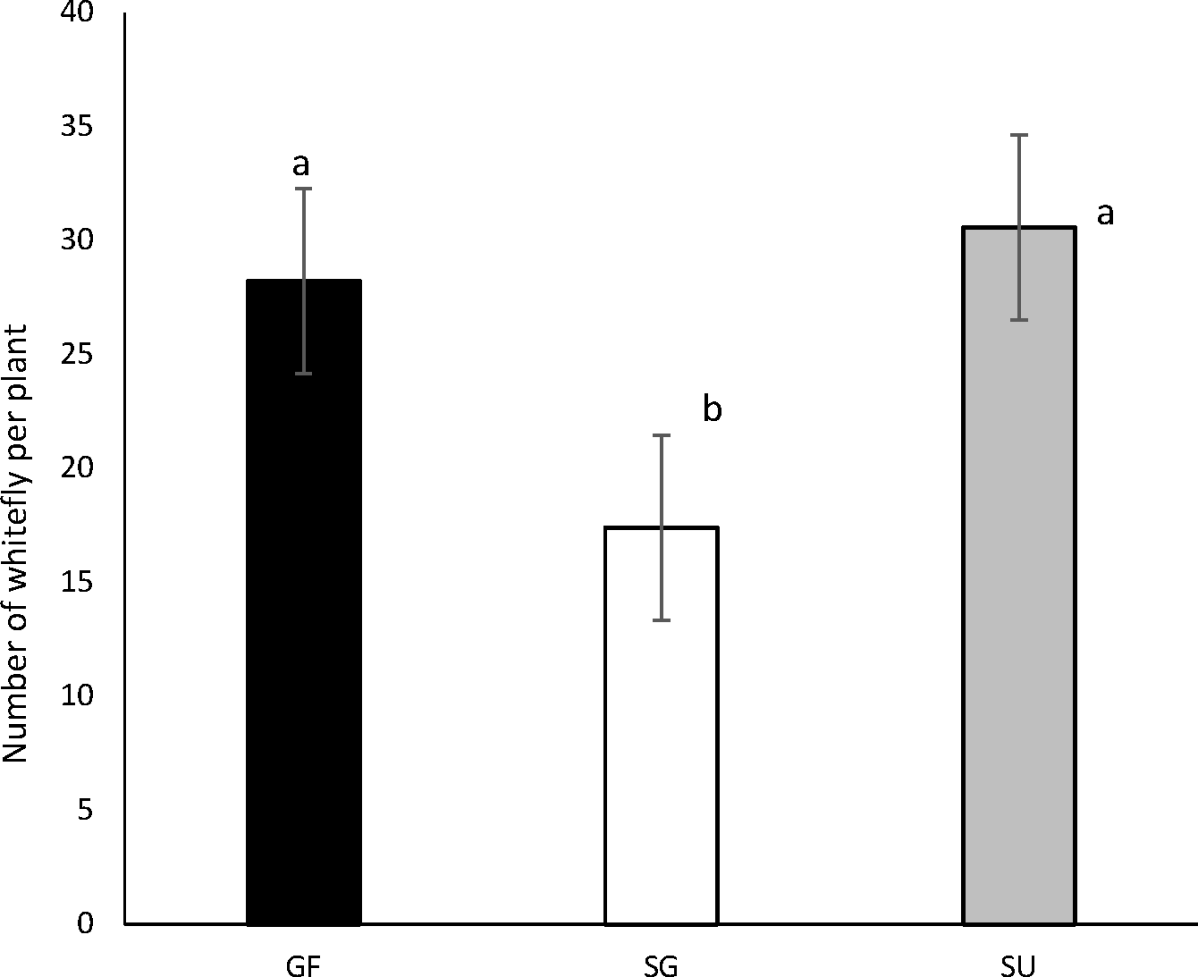
Average abundance of *B*. *tabaci* adults recorded on the top five leaves of 30 plants from sites in each of the main agro-ecological zones of Central African Republic. Vertical lines represent 95% confidence intervals, whilst means followed by the same letter are not significantly different (Generalized Tukey’s all pairwise comparisons test at P < 0.05). GF—Guinean Forest Zone; SG—Sudano-Guinean Zone; and SU—Sudano Ubanguian Zone.

*B*. *tabaci* adults were most abundant in the SU and GF zones (> 17 adults per plant). Whiteflies were significantly (P<0.005) more abundant in the SU (30.56±0.33) and GF (28.21±0.20) zones (Fig 1). There were no significant differences between SU (30.56±0.33) and GF (28.21±0.20) zones (Fig 1). In all cassava sampled fields CMD symptoms were observed at least on one cassava plant, which meant that the prevalence of CMD was 100%.

### Sequence analysis

#### Tests for neutrality

Tests for neutrality on the SSA1-SG1data subset were significant (Tajima’s D = -2.45317; Fu and Li’s F* = -4.05475; D* = -3.92597, all with P < 0.05), although the equivalent test results for SSA1-SG2 or MED were not significant, (MED: Tajima’s D = -1.48233; Fu and Li’s F* = -1.63609; D* = -1.48356; SSA1-SG2: Tajima’s D = -1.53850; Fu and Li’s F* = -1.72045; D* = -1.57025; all at P > 0.05). Significant values obtained for these criteria for SSA1-SG1 could be indicative either of the action of selection on this population or of demographic expansion. To untangle both hypotheses, the McDonald-Kreitman test, which is a robust measure of demographic changes, was performed. This test did not give any evidence for departure from neutrality (Fisher’s exact test, P-value = 0.09). We consequently assume that the SSA1-SG1 population has undergone an important growth of size. This was confirmed by the ‘‘R2” test of Ramos-Onsins and Rozas [31] (R2 = 0.0906, P-value = 0.03).

#### Phylogenetic analysis

**Fig 2:**
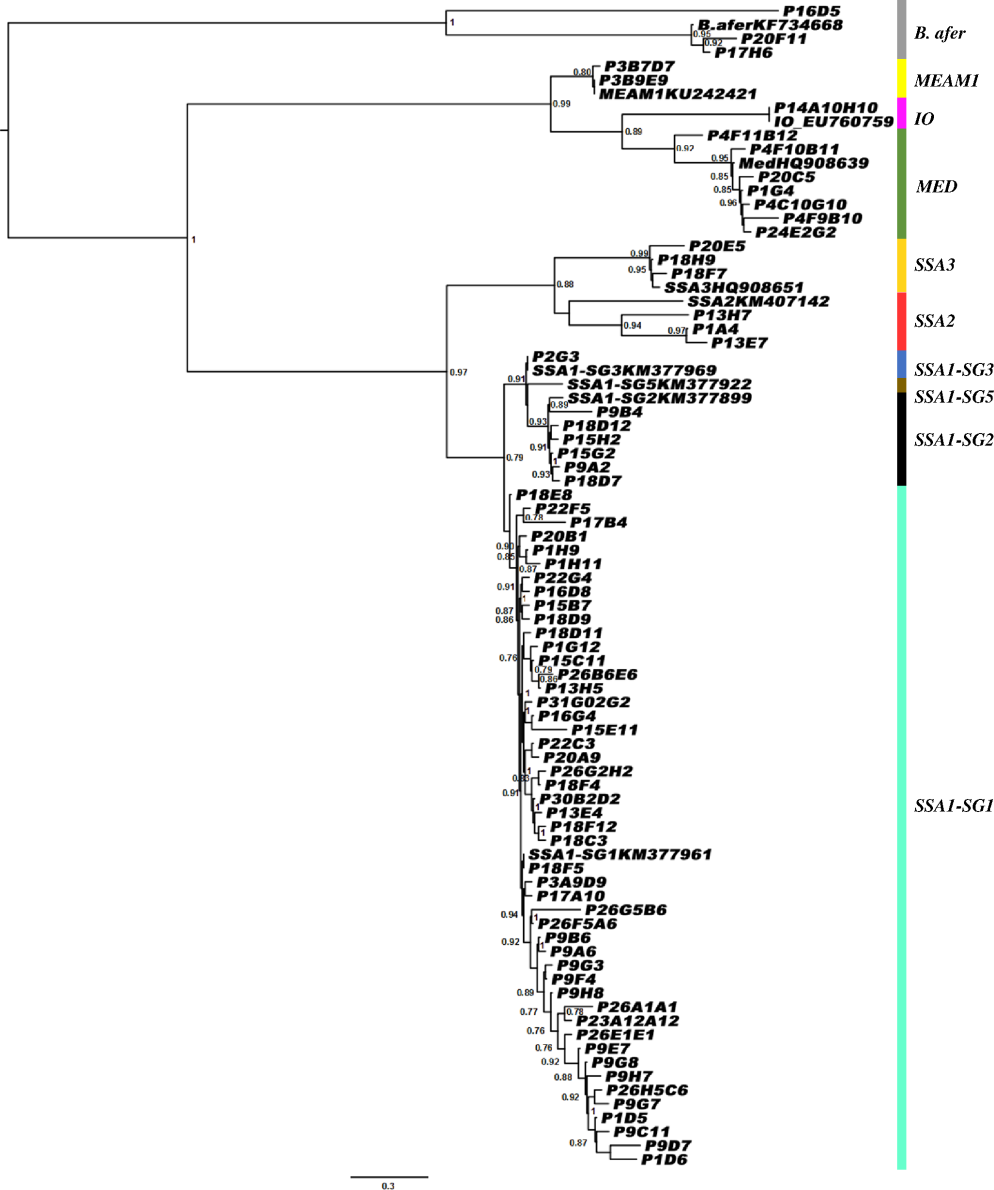
Phylogenetic tree indicating the relationships between the DNA sequences of 504 bp of the mitochondrial COI of *B*. *tabaci* for 74 unique sequences (see S1 Table) and those of a representative sampling of publicly available close sequences (Table 1). The tree was constructed using an approximate maximum likelihood phylogeny. Branch support was calculated by Shimodaira-Hasegawa-like local branch support (SH-like test).

There was considerable diversity among *B*. *tabaci* populations sampled, both between and within crop plants that were colonized (S1 Table). Phylogenetic analyses revealed six putative species of the species complex of *B*. *tabaci*, which included: sub-Saharan species/genetic group (SSA1-SG1, SSA1-SG2, SSA1-SG3 and SSA3), Mediterranean (MED), Indian Ocean (IO), Middle East Asia-Minor 1 (MEAM1), and another cassava species–*Bemisia afer* Priesner & Hosny (Fig 2). SSA1-SG1 sequences were the most numerous (n = 536 out of the 587 sequences analyzed) and exhibited the highest haplotype diversity (n = 48). Most of the SSA1-SG1 haplotypes (n = 370) clustered with Ugandan SSA1-SG1 (Mukono8-4 (KM377961) 100% nt identity) (Table 2) [38]. A very divergent SSA1 sequence (96% nt identity) was found (P2G3) which was closely related to SSA1-SG3. Twenty-seven individuals split into three different haplotypes were identified as SSA3 with the closest relative from GenBank being 5_Imo_state_SSA3 (Table 2). Another group comprised three haplotypes (n = 11), and was in the species group previously referred to as SSA2 [39], which was originally described from Uganda as Ug2 [40] (Fig 2). Fifty-nine individuals of non-cassava-colonizing putative species were also recorded. These included: IO (n = 1), MED (n = 51) and MEAM1 (n = 7). The MED species group individuals were found to be closely related to the B2.6 (HQ908639) [41] sequence described from Burkina Faso (100% nt identity) (Table 2).

**Table 2:** Result of the blast algorithm of GenBank when comparing the most frequent haplotypes from CAR to the worldwide diversity.

Species or putative species	Closest relative	Sequence name and accession number	Country
	(nt identity %)		
*Bemisia afer* (P17H6)	99–100	*Bemisia afer* (KF734668)	NA
SSA1-SG1 (P18F5)	100	Mukono8-4 (KM377961)	Uganda
SSA2 (P13H7)	99	Mukono1-7 (KM407142)	Uganda
SSA3 (P18H9)	100	NG_Owerri-5 (KM377923)	Nigeria
SSA3 (P18F7)	99	NG_Owerri-5 (KM377923)	Nigeria
SSA1-SG2 (P18D12)	99–100	DC105-2 (KF425628)	DR.Congo
SSA1-SG3 (P2G3)	100	UG_Ssanji (KM377969)	Uganda
MED (P1G4)	99–100	B2.6 (HQ908639)	Burkina Faso
MEAM1 (P3B9E9)	99–100	TO26 (KU242421)	Brazil
Indian Ocean (P14A10H10)	100	Reunion4 (EU760759)	Réunion island

#### Minimum spanning network analysis

**Fig 3:**
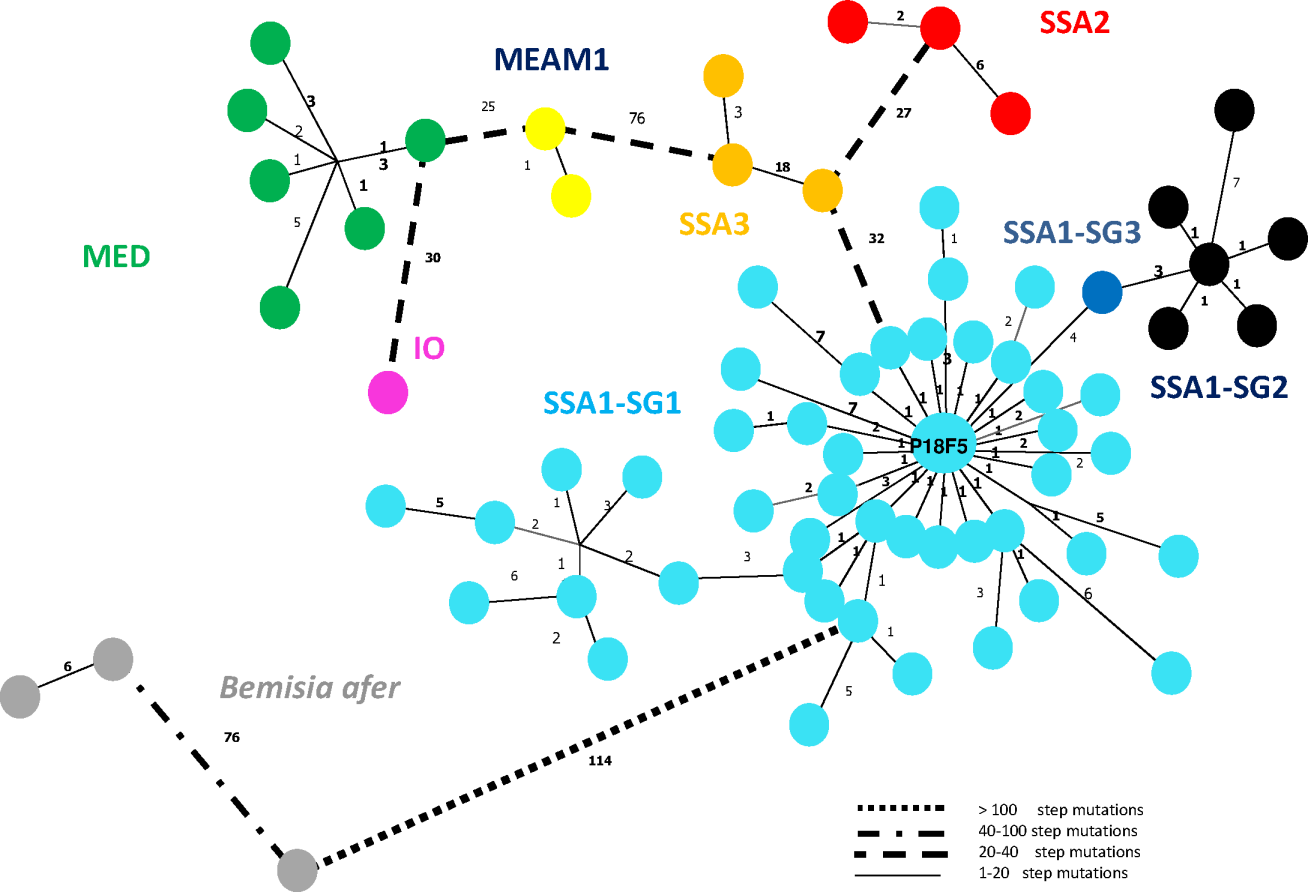
Minimum spanning network for *B*. *tabaci* SSA1, SSA2, SSA3, MEAM1, MED, Indian Ocean and *B*. *afer* species sampled from cassava crops and weeds in Central African Republic. Plain and dashed lines between circles represent mutational steps. Haplotypes with the same colored circle were assigned to the same genetic cluster.

**Fig 4:**
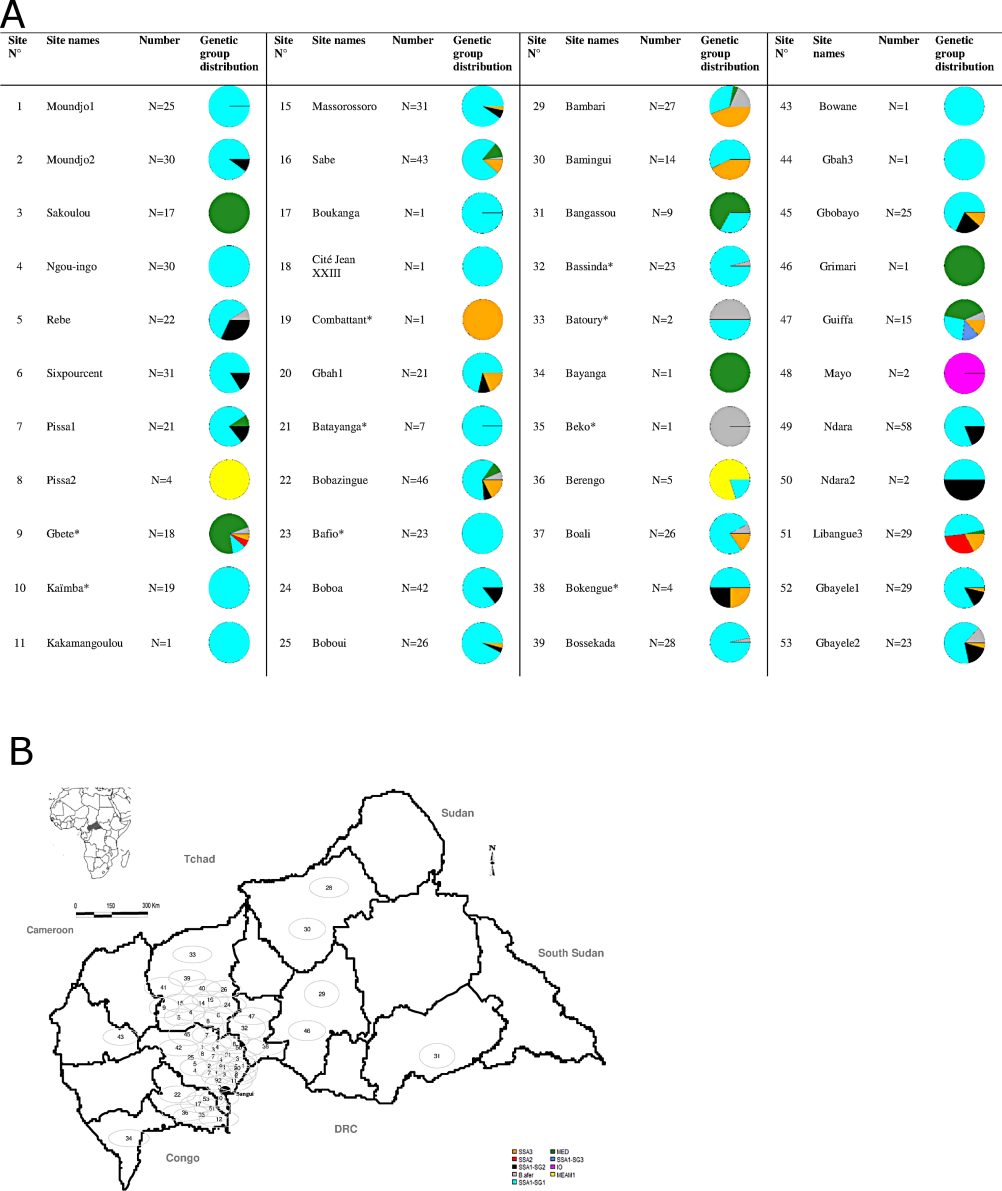
Distribution of *B*. *tabaci* putative species and genetic groups at the sampled sites in Central African Republic using 898 individuals. Each circle represents a site with a number referenced in the table below and each color corresponds to a genetic group.

A minimum spanning network was assembled to complement the phylogenetic analysis by illustrating the mutational steps between sequences (Fig 3). For the mtCOI sequences and the level of diversity analysis, 74 unique haplotypes were recognized from the full dataset of 587 sequences, and these were used in the network analysis (Table 1). A GoeBurst analysis was performed and a diagram based on a minimum spanning tree for all the unique haplotypes was drawn. Seven major haplotype groups corresponding to the species groups and several smaller satellite complexes were observed (SSA1-SG1, SSA1-SG2, SSA1-SG3, SSA2, SSA3, MED, IO, MEAM1 and *B*. *afer)*. The SSA1-SG1 group exhibited the highest level of haplotype diversity (n = 47) and signs of most mutational steps, although there was a single dominant haplotype (P18F5, n = 370). The MED group showed less diversity and fewer mutational steps with a dominant haplotype (P1G4). The mitochondrial sub-group SSA1-SG3 (P2G3, P21A2F2) was found in only one locality out of the 54 (Fig 4 and, S1 Table). This haplotype group was separated from SSA1-SG2 by only 3 step mutations. SSA1-SG2 was represented by 7 haplotypes. SSA3 haplotypes were separated from the SSA1 group by at least 32 mutational steps (Fig 3). Four other haplotype groups were observed, three of them from non-cassava whitefly species (IO, MED and MEAM1) and one from cassava-colonizing *B*. *afer*, which was the most distant from the *B*. *tabaci* species haplotype groups (114 step mutations).

### Occurrence and distribution of *Bemisia tabaci* haplotypes and species groups on host plants in CAR

Results demonstrated a geographic and host plant distribution of *B*. *tabaci* genetic group. The predominant genetic group in all geographic regions was SSA1-SG1. MEAM1 and IO were observed only in southern regions. MED individuals were observed in western and eastern regions.

**Fig 5:**
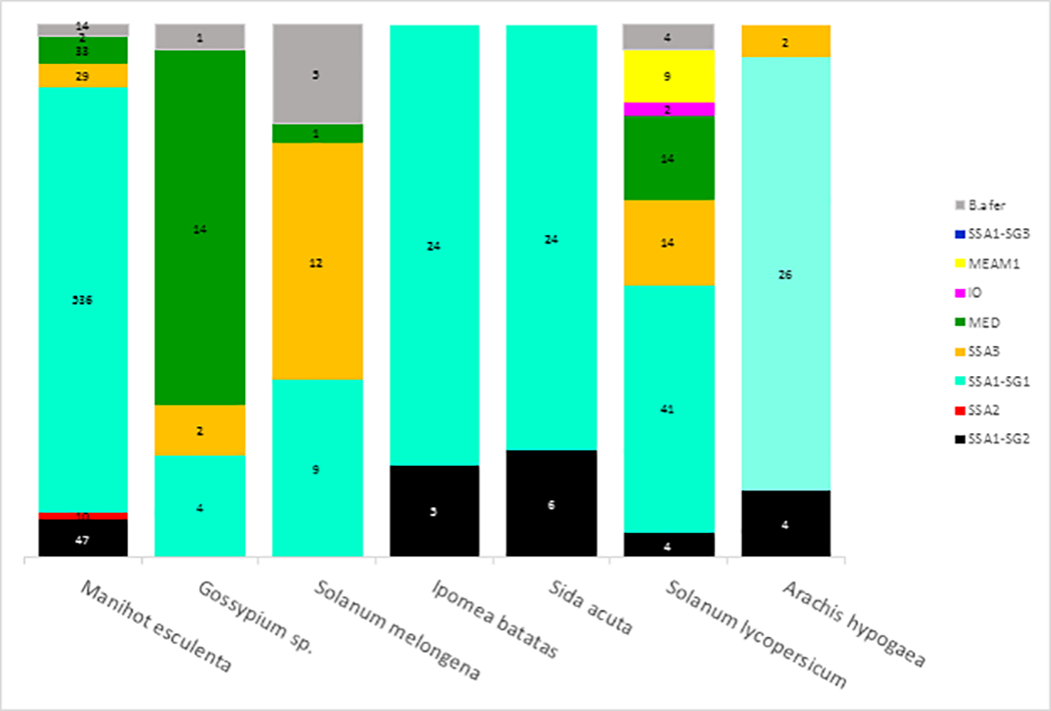
Distribution of 898 individuals of *B*. *tabaci* putative species and genetic groups on sampled host plants at sites in Central African Republic.

Our sampling mainly targeted cassava fields (78% of the whole dataset, and 51 sites out of 54). These samplings were made all over CAR and on eight different cassava cultivars. The second most sampled host plant was tomato, representing 10% of our sequences and 16/54 sites (Figs 4 and 5). Using the complete set of sequences (898), SSA putative species were dominant on cassava (93%) with the sub-group SSA1-SG1 occurring most frequently (79.9%). Within SSA1-SG1, the dominant haplotype P18F5 was found on all host plants (S1 Table, Figs 2–5). Nevertheless, this haplotype was not found on cassava in the sampling of 2007–2008 (although only 9 sites out of 54 were sampled during this period and 98 sequences processed). Other SSA putative species were found on all other hosts sampled, but in lower numbers (n = 177, representing 19.7%). SSA3 was found on cassava (n = 29, representing 4.3% of cassava samples), cotton, eggplant, groundnut and tomato. This putative species was the second most frequent in the dataset (n = 59, representing 7% of the whole dataset; Fig 5). The non-cassava putative species MED, MEAM1 and IO, were found on the other sampled plants (except on sweet potato, groundnut and the weed *S*. *acuta* where only SSA putative species were found). Tomato was the host plant on which MEAM1, IO and MED were most frequent, although SSA1-SG1 occurred more commonly than all three non-cassava putative species on these plants. *B*. *afer* was found sporadically on all host plants except sweet potato and the weed *S*. *acuta*. Most of the putative species of the *B*. *tabaci* species complex were found on tomato, including: SSA1 (-SG1 and -SG2), SSA3, MED, IO, MEAM1 and *B*. *afer*. From the 54 sampled sites, SSA1-SG1 was the dominant genotype (49/54) and, SSA1-SG2 (20/54) was always found in sympatry with SSA1-SG1. The same was found for SSA3, although this occurred less frequently, at eight sites, and it was less abundant (34/702) on cassava (Figs 4 and 5, S1 Table). Our data reveal that P18F5 occurred in all agroecological and geographical areas of the CAR in 2013.

## Discussion

### Whitefly abundance

A high abundance of adult whiteflies (> 17/plant) was recorded in the three agro-climatic zones sampled (SU, GF, SG), contrasting with the abundance values recorded in 2007 (1.5/plant) by Zinga and colleagues [13] and in Uganda during the severe CMD epidemic of the 1990s (7.1/plant) [23]. Legg [42] used a mean whitefly abundance value of ‘5’ as an arbitrary dividing line between countries or regions with low and high whitefly abundance. Using this definition, CAR has moved from the low to the high abundance category between 2007 and the time of the current study. Indeed, our results provide strong evidence that CAR is facing an upsurge in populations of the *B*. *tabaci* vector of cassava viruses and that the situation has changed recently (i.e between 2007 and 2013).

### Host plant association

The current study examined 898 *B*. *tabaci* individuals collected from cassava fields and associated crops in 54 sites spread throughout CAR (Table 1, Fig 5). Three sub-Saharan Africa putative species and three of their genetic sub-groups [SSA1 (-SG1, -SG2, -SG3); SSA2 and SSA3] plus three other non-cassava putative species of the *B*. *tabaci* complex (IO, MED and MEAM1) were found on seven host plants sampled. The SSA group [SSA1 (-SG1, -SG2, -SG3); SSA2, SSA3] was mostly found on cassava (n = 624, 93%), but also occurred frequently on other sampled plants (n = 177, 78% of sequences obtained from whiteflies on non-cassava plants). The exception was cotton, where MED was the predominant species. The non-cassava putative species IO and MEAM1 were only found on non-cassava crops.

Our results show a close association between *B*. *tabaci* putative species and host plants, as reported elsewhere [18, 39, 43, 44]. In addition, the results were also congruent with the reported occurrence from East Africa of SSA1-SG1 on a diversity of crop and weed host plants [45]. MED was the only *B*. *tabaci* putative species to be recorded from cotton in CAR. Similar results have been reported elsewhere, where MED (Q and ASL biotypes) were found on cotton [46]. The presence of whitefly on crops was also reported in Senegal on tomato for MEAM1 and MED [47]. The occurrence of four putative *B*. *tabaci* species on cassava in CAR (SSA1, SSA2, SSA3 and MED) highlights the diversity of *B*. *tabaci* there. These results are comparable with those reported in southern and East Africa where the same species were found, albeit in different proportions [18, 40]. Whilst MED, IO and MEAM1 occurred on a diverse group of annual crop and weed hosts, we only found them in three cases on cassava. Indeed, previous studies involving transfer of *B*. *tabaci* individuals between crop host plants have shown that non-cassava species were unable to reproduce on cassava [48–50]. SSA3, SSA2, SSA1-SG1, -SG2 and -SG3, which are considered to be cassava-colonizers, were also recorded on most of the other host plants sampled, although no nymphs were collected, which would have given an indication that the genotypes were successfully colonizing those plants. However, it has been demonstrated in Uganda that cassava-colonizing genotypes have a broader host range than was initially assumed [45], and this seems also to be the case in CAR. The infrequent occurrence of non-SSA putative species on cassava suggests that the small number of individuals recorded may have been visitors rather than residents. Sampling of late-stage nymphs for diagnostic sequencing could clarify this, since their presence on cassava would indicate successful colonization.

### Genetic diversity of *Bemisia tabaci*

Although we identified eight genetic groups within the *B*. *tabaci* samples sequenced, there was one group (SSA1-SG1) that predominated (74% out of the 898 dataset), and roughly half of these were from a single haplotype (P18F5, n = 370 representing 55.7%). Furthermore, this haplotype was not found in 2007–2008 (but only 9 sites out of 54 were sampled), despite the dominance of SSA1-SG1 with several different haplotypes (n = 10). In view of the predominance of this putative species on cassava, it might be suggested that this is the group mainly responsible for the spread of cassava viruses in CAR and the increased incidence of the disease that they cause. It may also be possible that the upsurge in *B*. *tabaci* abundance noted in recent years is associated with the pre-eminence of this group and the specific haplotype, as has been postulated for neighboring parts of East and central Africa [19]. The predominant SSA1-SG1 haplotype recorded from this study had 100% sequence identity with an SSA1-SG1 haplotype from Uganda [Mukono8-4 (KM377961)] (Table 2). Within the haplotype network the dominant haplotype was surrounded by a high diversity of haplotypes with few mutational steps, suggesting that the upsurge of SSA1-SG1 in CAR could be the result of a recent new introduction of this invasive haplotype, also suggested by its absence in the sampling performed in 2007–2008. Furthermore, negative and significant values obtained for the neutrality tests performed on this group, plus the demographic tests indicated a signal of demographic expansion for SSA1-SG1. Nevertheless, the occurrence of other smaller satellite SSA1-SG1 haplotype groups, the high overall diversity of these haplotypes, and their preeminence both in 2007–2008 and 2013 suggest that although SSA1 is likely to be the predominant and resident species group, there is evidence that haplotype P18F5 is a recent invasive population which has given rise to the upsurge in abundance in recent years. In East and Central Africa, SSA1-SG1 is often the group found where *B*. *tabaci* populations are abundant on cassava. Consequently, it has been associated with the spread of the cassava viruses causing severe CMD and CBSD in this region. In view of the apparent increased abundance of *B*. *tabaci* on cassava in CAR in recent years, coupled with the high incidences of CMD that have been recorded in this study and in [14], it seems likely that SSA1-SG1 may be playing a similar role here. Molecular studies of *B*. *tabaci* have provided evidence for a substantial degree of polymorphism between populations from distinct geographic regions [49, 51]. Results obtained from the current study may partially explain the cause of the outbreak of whiteflies in CAR on cassava. Although SSA2 was the putative species earlier associated with the spread of severe CMD in Uganda [40], super-abundant populations were shown in subsequent years to be dominated by SSA1-SG1. Data from the current study indicate that a similar change may have occurred in CAR, although the lack of any earlier data on *B*. *tabaci* putative species distribution and their haplotype diversity in CAR, means that it is not possible to confirm this. This highlights the importance of regular sampling *B*. *tabaci* populations in order to determine patterns of population change.

## Conclusions

Characterizing *B*. *tabaci* populations occurring on cassava, as well as other crop and weed hosts has led to a better understanding of the association between particular *B*. *tabaci* genotypes and their host plants in CAR. A high level of genetic diversity was revealed, although cassava-colonizing populations were predominantly SSA1-SG1 and of one specific haplotype (P18F5)–identical to a haplotype previously recorded from cassava in Uganda. Population genetics indicators applied to the whitefly sequence data obtained through this study suggest that SSA1-SG1 populations have undergone a recent expansion. The upsurge in populations of these cassava-colonizing whiteflies in CAR is likely to be the cause of the increase in CMD incidence observed between 2007 and 2013. Elevated populations of *B*. *tabaci* in CAR will additionally pose a significant threat in the near future for the spread of the other major virus disease affecting cassava in Africa, which is cassava brown streak disease. This has already been reported from neighboring Democratic Republic of Congo [10], and is expanding its range westwards [12]. These developments highlight the importance of developing and disseminating effective whitefly management strategies.

## Supporting information

S1 Table *B*. *tabaci* mtCOI sequences used to construct the phylogeny and haplotype network. Sequences presented were all of 504 bp and represent 587 samples obtained from Central African Republic. Nb: Number of each individual haplotype.
(DOCX)

## Abbreviations

COI: Cytochrome oxidase subunit I gene
mtDNA: mitochondrial DNA
