# Supplementary material for: Genetic diversity of Bemisia tabaci species colonizing cassava in Central African Republic characterized by analysis of cytochrome c oxidase subunit I
Source: PLoS One. 2017 Aug 15;12(8):e0182749. doi: 10.1371/journal.pone.0182749 (PMC5557543; doi:10.1371/journal.pone.0182749)
Supplement: S1 Table — Sequences presented were all of 504 bp and represent 587 samples obtained from Central African Republic. Nb: Number of each individual haplotype. (DOCX) [file pone.0182749.s001.docx]

Supplementary Table 1*. Bemisia tabaci* mtCOI sequences used to construct the phylogeny and haplotype network. Sequences presented were all of 504 bp and represent 587 samples obtained from Central African Republic. N: Number of each individual haplotype.

| **Host plant** | **Cassava variety** | **Sequence name** | **N** | **Putative**  **species name** | **Accession Number** | **Date** |
| --- | --- | --- | --- | --- | --- | --- |
| Eggplant |  | P1A4 | 1 | MED | LT707467 | 09/02/2013 |
| Cassava | Gobou | P1G4 | 28 | MED | LT707463 | 09/02/2013 |
| Cassava | Gobou | P4C10G10 | 2 | MED | LT707437 | 10/02/2013 |
| Tomato |  | P20C5 | 2 | MED | LT707458 | 27/03/2013 |
| Cassava | Gobou | P24E2G2 | 2 | MED | LT707451 | 10/02/2013 |
| Cassava | Gobou | P4F10B11 | 4 | MED | LT707436 | 10/02/2013 |
| Eggplant |  | P4F11B12 | 3 | MED | LT707435 | 10/02/2013 |
| Cassava | Gobou | P4F9B10 | 1 | MED | LT707434 | 10/02/2013 |
| Tomato |  | P14A10H10 | 1 | IO | LT707490 | 11/03/2013 |
| Groundnut |  | P18F7 | 1 | SSA3 | LT707469 | 20/03/2013 |
| Cassava | Gobou | P18H9 | 27 | SSA3 | LT707468 | 20/03/2013 |
| Cassava | Bandakpa | P13E7 | 9 | SSA2 | LT707493 | 09/03/2013 |
| Cassava | Bandakpa | P13H7 | 2 | SSA2 | LT707491 | 09/03/2013 |
| Tomato |  | P20E5 | 2 | SSA3 | LT707457 | 27/03/2013 |
| Tomato |  | P3B7D7 | 1 | MEAM1 | LT707439 | 10/02/2013 |
| Tomato |  | P3B9E9 | 6 | MEAM1 | LT707438 | 10/02/2013 |
| Cassava | Bandakpa | P17H6 | 6 | *Bemisia afer* | LT707479 | 19/03/2013 |
| Cassava | Bandakpa | P16D5 | 2 | *Bemisia afer* | LT707484 | 17/03/2013 |
| Cassava | Bandakpa | P20F11 | 1 | *Bemisia afer* | LT707456 | 27/03/2013 |
| Cassava, Eggplant, groundnut | Bandakpa | P18F5 | 370 | SSA1-SG1 | LT707470 | 20/03/2013 |
| Cassava | Bandakpa | P26H5C6 | 1 | SSA1-SG1 | LT707444 | 25/04/2008 |
| Cassava | Bandakpa | P30B2D2 | 12 | SSA1-SG1 | LT707442 | 28/06/2008 |
| *Sida acuta* |  | P9F4 | 10 | SSA1-SG1 | LT707426 | 22/02/2013 |
| Cassava | Bandakpa | P20B1 | 1 | SSA1-SG1 | LT707459 | 27/03/2013 |
| Groundnut |  | P18F4 | 1 | SSA1-SG1 | LT707471 | 20/03/2013 |
| Cassava | Folbagessé | P26G2H2 | 1 | SSA1-SG1 | LT707446 | 25/04/2008 |
| Groundnut |  | P18E8 | 1 | SSA1-SG1 | LT707473 | 20/03/2013 |
| Cassava | Folbagessé | P22F5 | 1 | SSA1-SG1 | LT707454 | 10/02/2013 |
| Tomato |  | P20A9 | 1 | SSA1-SG1 | LT707460 | 27/03/2013 |
| Cassava | Folbagessé | P26E1E1 | 1 | SSA1-SG1 | LT707448 | 25/04/2008 |
| Cassava | Folbagessé | P18C3 | 1 | SSA1-SG1 | LT707478 | 20/03/2013 |
| Groundnut |  | P18D11 | 3 | SSA1-SG1 | LT707477 | 20/03/2013 |
| Cassava | Folbagessé | P1H11 | 1 | SSA1-SG1 | LT707462 | 09/02/2013 |
| Groundnut |  | P18F12 | 4 | SSA1-SG1 | LT707472 | 20/03/2013 |
| Cassava | Folbagessé | P26F5A6 | 2 | SSA1-SG1 | LT707447 | 25/04/2008 |
| Cassava | Folbagessé | P31G02G2 | 1 | SSA1-SG1 | LT707441 | 01/07/2008 |
| Eggplant |  | P22C3 | 2 | SSA1-SG1 | LT707455 | 10/02/2013 |
| Cassava | Gboyo | P16G4 | 1 | SSA1-SG1 | LT707482 | 17/03/2013 |
| Cassava | Gboyo | P1H9 | 2 | SSA1-SG1 | LT707461 | 09/02/2013 |
| Cassava | Gboyo | P15E11 | 1 | SSA1-SG1 | LT707487 | 15/03/2013 |
| Cassava | Gboyo | P22G4 | 2 | SSA1-SG1 | LT707453 | 10/02/2013 |
| *Sida acuta* |  | P9G3 | 1 | SSA1-SG1 | LT707425 | 22/02/2013 |
| Sweet potato |  | P9B6 | 1 | SSA1-SG1 | LT707430 | 22/02/2013 |
| *Sida acuta* |  | P9C11 | 1 | SSA1-SG1 | LT707429 | 22/02/2013 |
| Cassava | Bawakou | P26A1A1 | 1 | SSA1-SG1 | LT707450 | 25/04/2008 |
| Cassava | Bawakou | P9H8 | 1 | SSA1-SG1 | LT707421 | 22/02/2013 |
| Sweet potato |  | P9H7 | 5 | SSA1-SG1 | LT707422 | 22/02/2013 |
| Cassava | Bawakou | P9G8 | 1 | SSA1-SG1 | LT707423 | 22/02/2013 |
| Sweet potato |  | P9G7 | 1 | SSA1-SG1 | LT707424 | 22/02/2013 |
| Sweet potato |  | P9D7 | 2 | SSA1-SG1 | LT707428 | 22/02/2013 |
| Cassava | Bawakou | P1D6 | 1 | SSA1-SG1 | LT707465 | 09/02/2013 |
| Cassava | Bawakou | P1D5 | 2 | SSA1-SG1 | LT707466 | 09/02/2013 |
| Cassava | Bawakou | P13E4 | 1 | SSA1-SG1 | LT707494 | 09/03/2013 |
| Cassava | Bawakou | P16D8 | 1 | SSA1-SG1 | LT707483 | 20/03/2013 |
| Groundnut |  | P18D9 | 1 | SSA1-SG1 | LT707474 | 20/03/2013 |
| Cassava | Bawakou | P3A9D9 | 1 | SSA1-SG1 | LT707440 | 10/02/2013 |
| Cassava | Bawakou | P23A12A12 | 1 | SSA1-SG1 | LT707452 | 06/06/2007 |
| Sweet potato |  | P9E7 | 2 | SSA1-SG1 | LT707427 | 22/02/2013 |
| Sweet potato |  | P9A6 | 1 | SSA1-SG1 | LT707432 | 22/02/2013 |
| Cassava | Kalangba | P26G5B6 | 1 | SSA1-SG1 | LT707445 | 25/04/2008 |
| Cassava | Kalangba | P17B4 | 1 | SSA1-SG1 | LT707480 | 19/03/2013 |
| Cassava | Kalangba | P17A10 | 2 | SSA1-SG1 | LT707481 | 19/03/2013 |
| Cassava | Kalangba | P15C11 | 3 | SSA1-SG1 | LT707488 | 15/03/2013 |
| Cassava | Kalangba | P13H5 | 1 | SSA1-SG1 | LT707492 | 09/03/2013 |
| Cassava | Kalangba | P1G12 | 1 | SSA1-SG1 | LT707464 | 09/02/2013 |
| Cassava | Kalangba | P26B6E6 | 1 | SSA1-SG1 | LT707449 | 25/04/2008 |
| *Sida acuta* |  | P9B4 | 2 | SSA1-SG2 | LT707431 | 22/02/2013 |
| Cassava | Laka | P15H2 | 2 | SSA1-SG2 | LT707485 | 15/03/2013 |
| Cassava | Laka | P15G2 | 22 | SSA1-SG2 | LT707486 | 15/03/2013 |
| Cassava | Laka | P18D12 | 1 | SSA1-SG2 | LT707476 | 20/03/2013 |
| Groundnut |  | P18D7 | 1 | SSA1-SG2 | LT707475 | 20/03/2013 |
| Cassava | Yabouta | P9A2 | 1 | SSA1-SG2 | LT707433 | 22/02/2013 |
| Cassava | Yabouta | P2G3 | 2 | SSA1-SG3 | LT707443 | 09/02/2013 |

Supplementary table 2. Sites coordinates and names of the 898 *Bemisia tabaci* sampled from Central African Republic. N: Number of samples per site.

| Site | N | Latitude | Longitude |
| --- | --- | --- | --- |
| **Moundjo1** | 25 | 04°55 994 | 018°41 818 |
| **Moundjo2** | 30 | 04°55 560 | 018°41 426 |
| **Sakoulou** | 17 | 04°07 646 | 018°15 712 |
| **Ngou-ingo** | 30 | 04°29 264 | 018°29 700 |
| **Rebe** | 22 | 04°56 414 | 018°41 726 |
| **Sixpourcent** | 31 | 04°50 247 | 018°01 729 |
| **Pissa1** | 21 | 04°12 334 | 018°23 007 |
| **Pissa2** | 4 | 04°07 529' | 018°14 534 |
| **Gbete** | 18 | NA | NA |
| **Kaïmba** | 19 | 04°29 264 | 018°29 700 |
| **Kakamangoulou** | 1 | 04°25 136 | 018°33 360 |
| **Kapou2** | 32 | 04°07 529' | 018°14 534 |
| **Gbah2** | 5 | 04°57 288 | 018°41 994 |
| **Sangara1** | 5 | 04°07 529' | 018°14 534 |
| **Massorossoro** | 31 | 04°10 914' | 018°22 772 |
| **Sabe** | 43 | 04°07 646' | 018°18 226 |
| **Boukanga** | 1 | 05°16 850' | 017°17 612 |
| **Cité Jean XXIII** | 1 | 04°25 137 | 018°33 360 |
| **Combattant** | 1 | NA | NA |
| **Gbah1** | 21 | 04°57 678 | 018°42 021 |
| **Batayanga** | 7 | NA | NA |
| **Bobazingue** | 46 | 04°07 646' | 018°18 226 |
| **Bafio** | 23 | NA | NA |
| **Boboa** | 42 | 04°10 913 | 018°22 766 |
| **Boboui** | 26 | 04°48 924 | 018°18 961 |
| **Bodengue** | 1 | 04°44 812 | 018°37 337 |
| **Gbango** | 2 | 04°39 946 | 018°33 920 |
| **Bakala** | 1 | 04°37 777 | 017°45 996 |
| **Bambari** | 27 | 06°56 628 | 019°48 768 |
| **Bamingui** | 14 | 07°56 524 | 020°11 791 |
| **Bangassou** | 9 | 05°50 927 | 021°57 160 |
| **Bassinda** | 23 | NA | NA |
| **Batoury** | 2 | NA | NA |
| **Bayanga** | 1 | 03°39 290 | 016°02 622 |
| **Beko** | 1 | NA | NA |
| **Berengo** | 5 | 04°07 646 | 018°18 226 |
| **Boali** | 26 | 04°53 268 | 018°06 299 |
| **Bokengue** | 4 | NA | NA |
| **Bossekada** | 28 | 05°49 071 | 018°03 927 |
| **Botili** | 20 | 04°30 758 | 018°31 509 |
| **Botini** | 1 | 04°17 174 | 017°12 137 |
| **Bounguende** | 24 | 04 °55 800 | 017°57 648 |
| **Bowane** | 1 | 04°36 847 | 017°49 318 |
| **Gbah3** | 1 | 04°45 540 | 018°36 315 |
| **Gbobayo** | 25 | 04°48 219 | 018°05 638 |
| **Grimari** | 1 | 05°44. 421 | 020°03 827 |
| **Guiffa** | 15 | 06°44 588 | 018°59 711 |
| **Mayo** | 2 | 04°56 764 | 018°41 844 |
| **Ndara** | 58 | 04°59 447 | 018°40 258 |
| **Ndara2** | 2 | 04°59 447 | 018°40 258 |
| **Libangue3** | 29 | 04°13 510 | 018°17 984 |
| **Gbayele1** | 29 | 04° 55 040 | 018°40 730 |
| **Gbayele2** | 23 | 04° 53 110 | 018°40 578 |
| **Bogomo** | 21 | 04° 07 647 | 018°21 980 |
